# Supplementary material for: MetaRibo-Seq measures translation in microbiomes
Source: Nat Commun. 2020 Jun 29;11:3268. doi: 10.1038/s41467-020-17081-z (PMC7324362; doi:10.1038/s41467-020-17081-z)
Supplement: Supplementary file 10 — Supplementary Data 7 [file 41467_2020_17081_MOESM10_ESM.zip › File2/Confidence_High_Taxonomy/84829_out.krona.html]

Javascript must be enabled to view this page.

members
magnitude
magnitudeUnassigned
count
unassigned
taxon
rank

84829\_out

6

2759
superkingdom
6

kingdom
6
3

SRS013687\_contig\_number\_32546SRS053356\_contig\_number\_23603SRS1041145\_contig\_number\_34725
4751

451864
2
subkingdom

phylum
2
4890

2
subphylum
147538

1
class
147541

1
subclass
451868

order
1
92860

1
suborder
715340

1
family
683158

104397
1
genus


SRS098571\_contig\_number\_49371
1
species
105696

147550
class
1

1
subclass
222544

order
1
5139

SRS023914\_contig\_number\_28410

phylum
1
4761

class
1
451435

1
order
642610

1
family
1396497

64515
1
genus

64517
1
species

SRS051031\_contig\_number\_36446
